# Physical Activity, Health-Related Quality of Life, and Stress among the Chinese Adult Population during the COVID-19 Pandemic

**DOI:** 10.3390/ijerph17186494

**Published:** 2020-09-07

**Authors:** Meiling Qi, Ping Li, Wendy Moyle, Benjamin Weeks, Cindy Jones

**Affiliations:** 1 School of Nursing, Shandong University, Jinan 250012, Shandong, China; meiling.qi@sdu.edu.cn; 2 Menzies Health Institute Queensland, Griffith University, Nathan Campus, Brisbane, QLD 4111, Australia; w.moyle@griffith.edu.au (W.M.); b.weeks@griffith.edu.au (B.W.); cjones@bond.edu.au (C.J.); 3 School of Nursing and Midwifery, Griffith University, Nathan Campus, Brisbane, QLD 4111, Australia; 4 School of Allied Health Sciences, Griffith University, Gold Coast Campus, Gold Coast, QLD 4222, Australia; 5 Faculty of Health Sciences & Medicine, Bond University, Gold Coast, QLD 4226, Australia

**Keywords:** physical activity, quality of life, perceived stress, COVID-19, Chinese adults

## Abstract

The COVID-19 pandemic poses a threat to global public health due to home confinement policies impacting on physical activity engagement and overall health. This study aimed to explore physical activity participation, health-related quality of life (HRQoL), and levels of perceived stress among Chinese adults during the COVID-19 pandemic. An online survey was conducted between 25 February and 15 March 2020. A total of 645 surveys were completed. Participants reported increased sedentary time from pre-COVID-19 period to the COVID-19 pandemic period (*p* < 0.05). Over 80% of the sample engaged in either low or moderate intensity physical activity. Participants’ average physical component summary score (PCS) and mental component summary score (MCS) for HRQoL were 75.3 (*SD* = 16.6) and 66.6 (*SD* = 19.3), respectively. More than half of participants (53.0%) reported moderate levels of stress. Significant correlations between physical activity participation, HRQoL, and levels of perceived stress were observed (*p* < 0.05). Prolonged sitting time was also found to have a negative effect on HRQoL (*p* < 0.05). During such periods of home confinement, public health strategies aimed at educating Chinese adults to enhance home-based physical activity may be necessary to maintain health on a population level.

## 1. Introduction

A local outbreak of pneumonia from an initially unknown cause was first detected in Wuhan, Hubei province China around December 2019 [1]. It rapidly affected over 20 provinces in China within a matter of weeks [2]. The World Health Organization (WHO) named the infectious coronavirus disease as COVID-19. In the middle of February 2020, almost 34,589 people in China were diagnosed with COVID-19. The Chinese government adopted extreme measures such as home confinement, lockdown of cities, extension of national holidays, and closure of schools nationwide to mitigate the outbreak of COVID-19 [3,4]. These measures, particularly home confinement, limited human mobility and effectively decreased the onset of new cases in mainland China [4]. Such restrictions were also found to have a negative impact on people’s daily living including social participation and life satisfaction [5,6].

The COVID-19 pandemic also poses a huge threat to global public health due to unprecedented individual and societal fear and anxiety, causing significant stress affecting health-related quality of life (HRQoL) [7]. HRQoL was reported to be associated with depression, anxiety, or negative general health in unmarried Iranian women during COVID-19 [8]. Such risks have also been identified in other pandemics. For example, severe acute respiratory syndrome (SARs) patients with increased levels of stress were at greater risk of experiencing negative mood and stress-related disorders [9]. A negative association between stress and quality of life can be anticipated but has yet to be formally evaluated during the COVID-19 pandemic. Additionally, researchers have demonstrated that there was a decrease in the level of outdoor physical activity and an increase in sedentary time during the COVID-19 pandemic as a result of home confinement [3,10]. A reduction in physical activity participation is known to contribute to stress levels, which is strongly associated with HRQoL [11,12].

Physical activity stimulates the production of endorphins to enhance mood and act as natural pain relievers and reduces the level of stress hormones such as adrenaline and cortisol [13]. Regular physical activity participation can also cause physiological adaptations in areas such as stroke volume, resting heart rate, blood pressure, and cardiac output, leading to improvements in cardiorespiratory and musculoskeletal performance that will promote individuals’ HRQoL [3]. Prolonged homestay could increase behaviors that lead to physical inactivity and contribute to stress. This, in turn, could lead to a sedentary lifestyle that may increase the risk of a range of chronic health conditions that adversely affect HRQoL [3]. For example, the limitation of daily activity has been found to significantly impair HRQoL among SARs patients in Hong Kong [14]. Thus, individuals who keep themselves physically active during the COVID-19 pandemic might present less stress symptoms and better levels of HRQoL. To date, however, there appears to be no study that has specifically investigated the levels of and the association between physical activity, HRQoL, and perceived stress in Chinese adults as a result of the COVID-19 pandemic. Understanding the above information can (a) corroborate the need for public health initiatives to increase physical activity participation and (b) inform the development of new home-based physical activity interventions for future use. Thus, the purpose of this study was to explore physical activity participation, HRQoL, and perceived stress among the Chinese adults during the COVID-19 pandemic.

## 2. Materials and Methods

### 2.1. Study Design

A cross-sectional online survey of ethnic Chinese was conducted. The study was conducted in accordance with the Declaration of Helsinki as established by the World Medical Association, and ethics approval for this study was gained from the Shandong University Human Research Ethics Committee (*2020-12-037*). Completion and submission of the online survey implied consent to participate in this study, which was declared to respondents at the commencement of the survey.

### 2.2. Participants

Ethnic Chinese adults (i.e., aged 18 years and over) living in the mainland of China were recruited for this survey study.

### 2.3. Procedure

Data collection took place between 25 February and 15 March 2020. Participants were invited to complete an online questionnaire that was administered via a free online Chinese survey platform (https://www.wjx.cn). Potential participants were recruited through the WeChat mobile instant text communication service that has the largest number of Chinese users compared to any other communication tool and is considered to be the most important social media platform among the Chinese population [15].

### 2.4. Measures

The survey sought information on participants’ (a) demographic characteristics (e.g., age, gender, education level, income, general physical activity participation frequency (i.e., low, medium and high frequency) [16] (pre-COVID-19) and sedentary time (hours per day) (pre- and during COVID-19), and relevant COVID-19 questions (i.e., knowledge of, and access to, COVID-19 information); (b) current physical activity participation; (c) HRQoL; and (d) perceived stress.

#### 2.4.1. Physical Activity

The International Physical Activity Questionnaire Short Form (IPAQ-SF)—Chinese version [17] was used to assess participants’ physical activity participation during the past 7 days. There are 2 types of IPAQ scores for data processing and analysis: a categorical and a continuous score. The categorical score classified participants into 3 physical activity intensity levels (i.e., low, moderate, and high). The continuous score is expressed as the metabolic equivalent task (MET-minutes per week) of energy expenditure. Participants’ sitting time (i.e., hours per day) was also recorded on the IPAQ-SF. Reliability for the IPAQ short version has been established among Chinese adults with intraclass correlation coefficients above 0.70 [18].

#### 2.4.2. Health-Related Quality of Life

The Chinese version of the SF-8 [19] was used to assess participants’ HRQoL. The SF-8 comprises of 8 items: physical functioning (PF), general health (GH), role physical (RP), bodily pain (BP), mental health (MH), social functioning (SF), vitality (VT), and role emotional (RE). The raw scores of each item were coded, weighted, and summed into 2 scores: physical component summary score (PCS) and mental component summary score (MCS), with higher scores indicating better quality of life. The SF-8 has satisfactory reliability and validity for use with the Chinese population (Cronbach’s α = 0.75) [19].

#### 2.4.3. Perceived Stress

The 10-item Perceived Stress Scale (PSS-10)—Chinese version [20] was used to assess participants’ perceived stress experienced across the past month. A score of 13 or less reflects low stress, with moderate and high perceived stress reflected by scores ranging from 14 to 26 and 27 to 40, respectively. PSS-10 has established reliability and validity for use with Chinese people (Cronbach’s α = 0.67–0.86) [21,22].

### 2.5. Statistical Analysis

Analysis of data was conducted using the Statistical Package for the Social Sciences (SPSS) version 26.0 [23]. Beside descriptive statistics using frequencies (i.e., percentages) for categorical variables and mean and standard deviations for continuous variables, associations between physical activity, HRQoL, and perceived stress with demographic characteristics were computed using Pearson’s r, paired *t*-test, and analysis of variance (ANOVA). The significant level was set at 0.05.

## 3. Results

### 3.1. Parcitipant Characteristics

**Figure 1:**
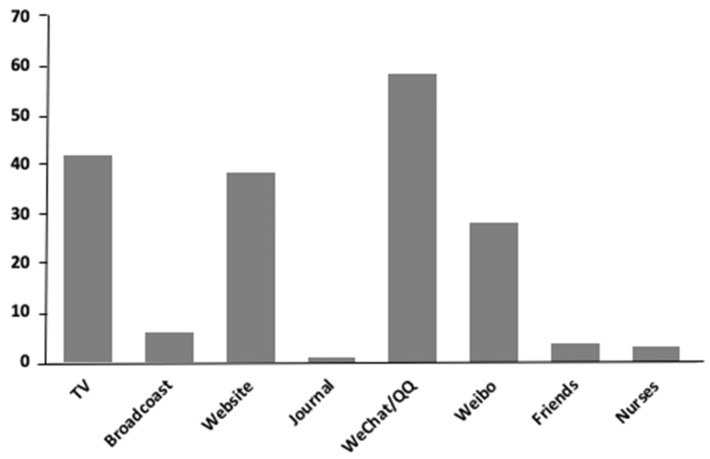
Mode of access to COVID-19 information (% respondents per item) (*n* = 645).

A total of 645 participants completed the survey (Table 1). Participants were aged between 18 and 66 years (*M* = 31.8; *SD* = 8.6). Among these participants, most were female (61.2%), had a balance between income and expenditure (63.7%), and lived in urban areas (70.1%). More than half of the participants were married (65.0%), with only 2.0% being divorced, widowed, or separated. More than 90% of the participants paid moderate or high levels of attention to information related to COVID-19. Participants reported various methods of access to COVID-19 information (Figure 1), including social apps (i.e., WeChat and QQ), television, and websites including Weibo (i.e., a Chinese microblogging website).

### 3.2. Parcitipant Physical Activity (PA) Particiption, Sedentary Time, HRQoL, and Stress

Findings for physical activity participation, sedentary time, HRQoL, and perceived stress are presented in Table 2. The majority of participants had undertaken medium (i.e., approximately 2–4 times per week; 49.3%) to high frequency (i.e., more than five times per week; 26.1%) of physical activity prior to the COVID-19 pandemic. However, 64.8% of the participants engaged in low amounts of physical activity (i.e., less than 600 MET-min/week) during the COVID-19 pandemic. Only a respective 18.0% and 17.2% of participants engaged in moderate and high levels of physical activity. Importantly, there was a significant increase in mean sedentary time from the pre-COVID-19 period (M = 5.4, *SD* = 2.9) to the COVID-19 pandemic period (M = 5.8, *SD* = 4.6), (*t* (644) = −2.6, *p* < 0.05). Participants’ average PCS and MCS scores for HRQoL were 75.3 (*SD* = 16.6) and 66.6 (*SD* = 19.3), respectively. Furthermore, more than half of the participants (53.6%) reported moderate levels of perceived stress during the COVID-19 pandemic.

### 3.3. Associations between Participant Characteristics and PA, HRQoL, and Stress

There are significant differences in physical activity participation between genders (*t* (345.6) = 3.8, *p* < 0.01) and living areas (*t* (426.9) = 2.6, *p* = 0.01), where people who are males and living in urban areas engaged in more exercise than those who are females and living in the suburbs (see Table 3). Participants who are married and possessed a TAFE/college degree and bachelor or postgraduate degree reported significantly lower levels of perceived stress than those unmarried (*p* = 0.03) and with a lower than high school degree (*p* < 0.01), respectively. Significant differences were also found in PCS scores (*f* (2, 642) = 3.9, *p* = 0.02) and *f* (2, 642) = 3.5, *p* = 0.03) and levels of perceived stress (*f* (2, 642) = 15.0, *p* < 0.01 and *f* (2, 642) = 3.2, *p* = 0.04) for the different family income groups and pre-COVID-19 physical activity history. Post-hoc tests reveal that participants who had sufficient income for expenditure and engaged in exercise very often pre-COVID-19 reported higher PCS scores and lower levels of perceived stress than those who had insufficient income (*p* < 0.05) and occasionally joined exercise before the COVID-19 pandemic (*p* = 0.02). Additionally, ANOVA with post-hoc tests revealed that participants who had insufficient income reported significantly lower MCS scores than those who had sufficient income (*p* < 0.01) and had a balance between income and expenditure (*p* < 0.01).

### 3.4. Associations between Sedentary Time, PA, HRQoL and Stress

As reflected in Table 4, During the COVID-19 pandemic, physical activity participation had a significant positive association with PCS scores (*p* = 0.002, *r* = 0.1) and a negative association with perceived stress levels (*p* = 0.002, *r* = −0.1). Thus, participants who engaged in more physical activity reported higher levels of HRQoL and lower levels of perceived stress. Perceived stress scores were negatively associated with PCS (*p* < 0.001, *r* = −0.4) and MCS (*p* < 0.001, *r* = −0.6), indicating that participants reported higher HRQoL when experiencing lower levels of perceived stress. Significant negative associations between average sedentary time during the COVID-19 outbreak and PCS scores (*p* < 0.05, *r* = −0.1) were found where participants who reported greater sedentary time experienced lower HRQoL.

## 4. Discussion

A better understanding of physical activity participation, HRQoL, and perceived stress in Chinese adults during the COVID-19 pandemic is worthy of the attention of researchers. This study extends our knowledge of Chinese adults’ physical activity levels, HRQoL, and perceived stress during the COVID-19 pandemic, which revealed that (a) the pandemic had negative effects on the levels of daily sitting time and physical activity participation, (b) the majority of participants reported moderate or high levels of perceived stress where stress was found to have a strongly negative association with people’s HRQoL during the pandemic, and (c) there were significant associations between levels of physical activity, HRQoL and perceived stress.

The sedentary activity findings of the current study are consistent with those of previous studies, indicating that participants reported significantly higher sedentary time during the COVID-19 pandemic. Qin et al. [10] demonstrated that almost 60% of Chinese adults had longer sedentary time during the COVID-19 pandemic (i.e., 4 h longer per day). Ammar et al. [24] also indicated that the international population from Europe, North-Africa, Western Asia, and the Americas had a sitting lifestyle increase from 5 to 8 h per day during the COVID-19 pandemic. This may be related to an increased use of social media (e.g., WeChat) to access relevant COVID-19 information and watching television because of the home confinement restriction when no work or business was conducted. Despite its statistical significance, only a small increase in means for the average sitting time during the COVID-19 period (0.4 h) was found. This could result from participants’ self-reported bias, since the majority of them might be sensitive to the COVID-19 pandemic due to the moderate or high levels of stress when completing the survey.

More than half of the participants in the current study reported moderate or high levels of perceived stress. Similar results were found in health professionals (i.e., almost 57%) during the SARs outbreak [25]. The high percentage of perceived stress might be associated with (a) worries about their own health and that of their family members, and (b) the spread of the virus which negatively affects daily living. Lam et al. [26] indicated that people from low-income households may be unwilling to spend their limited income on health-related goods or services. As a result, another potential reason for the reported stress may be related to family income during home confinement where the current findings showed that lower family income was associated with higher levels of stress. The current study also found that people with a bachelor or TAFE/college qualification had lower levels of perceived stress compared with those with only a high school qualification. Qi et al. [27] concluded that educated people may have relevant knowledge in accessing appropriate resources and selecting effective strategies to eliminate negative moods and manage their stress symptoms. Thus, relevant online educational programs may have the potential to reduce people’s levels of perceived stress during the COVID-19 pandemic.

Correlations between the quality of life and mental health have been shown in a recent study conducted by Zahra et al. [8]. Furthermore, income may be related to additional social factors (e.g., education and health literacy) and has also been previously associated with decreased quality of life in patients with SARs [28,29]. This study contributes to the literature demonstrating the negative impact of family income on HRQoL in adults during the COVID-19 pandemic. Apart from the above influencing factors, this current study found that stress had a strongly negative effect on HRQoL in Chinese adults during the COVID-19 pandemic. Whereas a study conducted by Tsang [30] that recruited 148 nurses did not show that stress had a negative impact on nurses’ quality of life during the SARs epidemic. Further studies are needed to confirm the association between stress and HRQoL during or after the COVID-19 pandemic. Evidence from a review reported several psychological perturbations and emotional disturbances such as stress and quality of life in SARs infected patients during the quarantine periods [31]. A recent study also reported that home confinement evoked a negative effect on mental wellbeing and emotional status because of experienced physical inactivity, social isolation as well as unemployment during the COVID-19 pandemic [32]. Importantly, sedentary behavior caused by the implementation of confinement policies to combat COVID-19 negatively impacted people’s quality of life in this current study. Therefore, it is critically important to promote people’s health and wellbeing by encouraging them to minimize sitting time and keep physically active in an effort to maximize HRQoL during the COVID-19 pandemic.

Home confinement has the potential to decrease people’s outdoor physical activity participation and increase their sedentary time [24,33]. With the continuing spread of COVID-19, people were advised to stay at home which may explain the lower levels of physical activity participation observed compared to the pre-COVID-19 period in this current study. Physical activity participation was found to be positively associated with HRQoL and negatively related to perceived stress. These statistics are supported by other similar studies where physically active people reported significantly higher HRQoL and lower levels of perceived stress [11,12]. Regular exercise has also been found to have the ability to reduce the severity of infections when people continued to remain active during the SARs period and they were more likely to survive compared with people who were more sedentary [34,35]. Thus, due to the restrictions of home confinement, appropriate home-based exercises (e.g., strength training, walking at home, lifting, and following online exercise) are recommended behavioral strategies for staying active at home to promote people’s overall health [3]. To do home-based exercise safely, people, particularly older adults, are encouraged to participate in home-based exercise according to the American College of Sports Medicine (ACSM) recommendations [36]. However, it is important that people are screened pre-exercise. For example, de Oliveira Neto et al. [37] reported that the screening of COVID-19 related symptoms (e.g., cough, difficulty of breathing, or fever) before exercise may help to identify those who can safely start an exercise program.

The strength of this study is the exploration of physical activity participation, HRQoL, and perceived stress in Chinese adults affected by the COVID-19 pandemic, and a relatively large sample completing the survey (*n* = 645). Recruitment was challenging as the WeChat tool was the only viable strategy to recruit participants due to home confinement during the COVID-19 pandemic. A limitation of the study is the self-reported data that is subject to a degree of participant bias and may be impacted by self-recall. Additionally, the impact of different types of physical activity and job as well as the presence of children on HRQoL and perceived stress, which were not determined in the study, could be considered in future studies.

## 5. Conclusions

This study suggests that physical activity participation, HRQoL, and perceived stress are significantly related among Chinese adults during the COVID-19 pandemic. Home confinement during the pandemic may have the potential to decrease Chinese people’s physical activity and sedentary sitting time. Prolonged sitting time was also found to have a negative effect on HRQoL. People with lower family income have higher levels of perceived stress. However, those who are educated may be better able to manage stress which is reflected by lower reported perceived stress. There is a need to translate the findings of this research in future home-based exercise intervention studies in order to improve participants’ physical activity participation and mental health during and after the COVID-19 pandemic.

## Figures and Tables

**Table 1:** Demographic characteristics of participants (*n* = 645).

Category	Mean (*SD*)	*n*	%
**Age (years)**	31.8 (8.6)		
**Gender**			
Female		395	61.2
Male		250	38.8
**Geographic classification**			
Suburban		193	29.9
Urban		452	70.1
**Marital status**			
Single		213	33.0
Married		419	65.0
Other (divorced, widowed, or separated)		13	2.0
**Education level**			
Less than high school		138	21.4
TAFE/College		155	24
Higher than bachelor’s degree		352	54.6
**Family income**			
Income insufficient for expenditure		71	11.0
Balance between income and expenditure		411	63.7
Income sufficient for expenditure		163	25.3
**Attention to COVID-19**			
Low		43	6.7
Moderate		241	37.3
High		361	56.0

SD, standard deviation; TAFE = Technical and further education; COVID-19, corona virus disease 2019.

**Table 2:** Descriptive statistics for PA, HRQoL, and stress scores (*n* = 645).

Category	Mean (SD)	*n* (%)
**PA participation frequency pre-COVID-19**		
Low (less than 1 days per week)		157 (24.3%)
Medium (2–4 times per week)		318 (49.3%)
High (more than 5 times per week)		170 (26.4%)
**Sedentary time (h/day) pre-COVID-19**	5.4 (2.9)	
**Sedentary time (h/day) during COVID-19**	5.8 (4.6) *	
**IPAQ scores (MET-min) during COVID-19**	850.0 (1567.3)	
**IPAQ (PA participation) during COVID-19**		
Low		418 (64.8%)
Moderate		116 (18.0%)
High		111 (17.2%)
**HRQoL during COVID-19**		
PCS	75.3 (16.6)	
MCS	66.6 (19.3)	
**Stress scores during COVID-19**	14.2 (6.2)	
**Stress level during COVID-19**		
Low stress		286 (44.3%)
Moderate stress		348 (53.6%)
High stress		13 (2.1%)

HRQoL, Health-Related Quality of Life; IPAQ, International Physical Activity Questionnaire; SD, standard deviation; COVID-19, corona virus disease 2019; PA, physical activity; PCS, physical component score; and MCS, mental component score. * significant difference (*p* < 0.05) between pre-COVID-19 and during COVID-19 data.

**Table 3:** Comparison between participant characteristics and PA, PCS, MCS, and stress (*n* = 645).

Participant Characteristics	PA		PCS		MCS		Stress	
	Mean (*SD*)	*p*	Mean (*SD*)	*p*	Mean (*SD*)	*p*	Mean (*SD*)	*p*
**Gender ^a^**								
Female	640.0 (1124.5)		75.9 (16.2)		67.6 (18.4)		14.1 (6.1)	
Male	1181.7 (2046.6)	0.000	74.3 (17.3)	0.24	65.1 (20.6)	0.12	14.3 (6.2)	0.67
**Geographic classification ^a^**								
Suburb	619.5 (1376.0)		77.0 (16.4)		67.3 (19.5)		14.7 (5.9)	
Urban	948.4 (1633.8)	0.01	74.6 (16.7)	0.09	66.4 (19.3)	0.59	13.9 (6.3)	0.13
**Marital status ^b^**								
Single	938.7 (1795.6)		76.9 (16.1)		65.6 (19.5)		15.0 (6.0)	
Married	797.6 (1453.4)		74.5 (16.8)		67.0 (19.2)		13.7 (6.2)	
Others	1083.2 (999.6)	0.49	74.5 (18.5)	0.22	70.7 (21.4)	0.53	15.5 (6.3)	0.03
**Educational level ^b^**								
Less than high school	856.2 (1450.6)		72.9 (18.1)		64.2 (20.8)		16.0 (6.2)	
TAFE/College	885.3 (1505.2)		76.4 (16.3)		67.0 (18.3)		13.6 (6.7)	
High than bachelor’s degree	832.0 (1640.4)	0.94	75.8 (16.1)	0.15	67.4 (19.1)	0.25	13.7 (5.8)	0.000
**Family income ^b^**								
Income insufficient for expenditure	755.0 (1703.9)		71.3 (17.1)		58.8 (18.3)		17.0 (6.1)	
Balance between income and expenditure	800.3 (1445.5)		75.0 (16.9)		66.4 (19.5)		14.4 (6.0)	
Income sufficient for expenditure	1016.4 (1784.6)	0.29	77.7 (15.6)	0.02	71.6 (18.3)	0.000	12.4 (6.1)	0.000
**PA participation frequency before COVID-19 ^b^**								
Rarely (less than 2 times per week)	233.0 (544.8)		75.0 (17.6)		66.4 (19.0)		15.0 (6.5)	
Occasionally (3–4 times per week)	585.2 (981.5)		74.0 (15.9)		66.7 (19.3)		14.1 (6.0)	
Very often (more than 5 times per week)	1915.1 (2377.7)	0.000	78.1 (16.7)	0.03	67.6 (19.8)	0.98	13.3 (6.1)	0.04

*SD*, standard deviation; PA, physical activity; PCS, physical component score; MCS, mental component score. Mean (*SD*) of PA (MET-minutes) reported based on the International Physical Activity Questionnaire (IPAQ) protocol. ^a^ The value was calculated with independent *t* tests; ^b^ the value was calculated with one-way analysis of variance (ANOVA). Significant level (*p* < 0.05).

**Table 4:** Association between sedentary time, PA, PCS, MCS, and stress (*n* = 645).

	Sedentary Time	PA	PCS	MCS	Stress
**Sedentary time**	---				
**PA**	−0.0	---			
**PCS**	−0.1 *	0.1 *	---		
**MCS**	−0.1	0.1	0.6 *	---	
**Stress**	−0.0	−0.1 *	−0.4 *	−0.6 *	---

PA, physical activity; PCS, physical component score; MCS, mental component score. * significant level (*p* < 0.05).
